# Weaving Regenerative Value Through Nature-Inclusive Stakeholder Engagement: Insights From Indigenous Businesses in the Sierra Nevada

**DOI:** 10.1177/00076503251407454

**Published:** 2026-02-02

**Authors:** Silvan Oberholzer

**Affiliations:** 1 University of St.Gallen, Switzerland; 2 HWZ University of Applied Sciences in Business Administration Zurich, Switzerland

**Keywords:** Indigenous business, nature-inclusive stakeholder engagement, regenerative organizing, Sierra Nevada de Santa Marta, stakeholder theory

## Abstract

Nature-inclusive stakeholder engagement, grounded in Western epistemologies and ontologies, embraces organizational relationships with nature entities considered as stakeholders. Although this construct has conceptual links to regenerative value creation, how it unfolds in an organizational setting remains unclear. This qualitative study informs nature-inclusive stakeholder engagement from a managerial perspective by exploring how Indigenous businesses of the Arhuaco (*Wíntukua*), Kogui (*Kággaba*), Wiwa (*Arzario*), and Kankuamo (*Kaku’chukwa*) peoples of the Sierra Nevada de Santa Marta, Colombia, consider nature relationships. The article relies on 27 semi-structured interviews with Indigenous business representatives, secondary literature (co-)authored by the Indigenous peoples of the Sierra Nevada, and observation data. The empirical findings provide insight into a nature-inclusive stakeholder engagement mindset, related interactions, and resulting regenerative value. Adopting Two-Eyed Seeing, the article proposes multidimensional nature-inclusive stakeholder engagement that embraces nature relationships through a relational stakeholder approach, wherein nature informs regenerative value creation as a guide.

Non-Western forms of organizing, such as the community-based Indigenous businesses of the Arhuaco (*Wíntukua*), Kogui (*Kággaba*), Wiwa (*Arzario*), and Kankuamo (*Kaku’chukwa*) peoples of the Sierra Nevada de Santa Marta (hereafter Sierra Nevada), Colombia, provide insights into regenerative value creation refined over millennia. Indigenous businesses are embedded in capitalist market structures but are created, self-identified, and accepted, owned, run, and controlled by Indigenous communities that rely on their worldviews, traditional knowledges, values, and cultures (Foley & Hunter, 2013; Peredo & McLean, 2010). These businesses focus on achieving multiple community-oriented goals in response to (neo)colonial oppression (Berkes & Adhikari, 2006; Peredo, 2023; Peredo & Chrisman, 2006), including promoting the strengthening and survival of their Indigenous cultures and worldviews, which are place-based (i.e., relational and tied to their territory or land, acting as epistemic agent; Ehrnström-Fuentes & Böhm, 2023). Underrepresented in emerging management and business research on regenerative organizing (Konietzko et al., 2023; Salmon et al., 2023), few studies have illustrated how Indigenous businesses worldwide contribute to regenerative value creation (Bellato et al., 2024; Peredo, 2019; Spiller et al., 2011). For creating such value, understanding human-nature stakeholder relationships, wherein non-human nature (hereafter referred to as nature) is considered as more than an environment, exploitable resource, asset, or waste repository (Arjaliès & Banerjee, 2024; Kortetmäki et al., 2023), but instead as a partner (Heikkinen et al., 2019), becomes vital.

Regenerative business contributes to the continued functioning and flourishing of society and nature through mutually beneficial stakeholder relationships that foster responsible consumption and production, extending beyond the fulfillment of human interests and needs (Hahn & Tampe, 2021; Muñoz & Hernandez, 2024). This style of business contrasts with the predominant Western business logic of take-make-use-waste, aimed at rapid economic growth based on profit maximization and, at best, embracing a minimal harm approach (Heikkurinen et al., 2021; Kearins et al., 2010). Characterized by “humans’ transactional and extractive relationships with nature” (Arjaliès & Banerjee, 2024, p. 29), which foster a human-nature disconnect and dualism (Ferns, 2025; Gualandris et al., 2024), this logic has caused irreversible environmental degradation that is increasingly disrupting societal systems, including business itself (Ehrnström-Fuentes et al., 2025; Intergovernmental Panel on Climate Change, 2023). In contrast, regenerative businesses “purposefully restore and regenerate degraded living ecosystems and deliberately build resilience in and improve the well-being of the communities relying on such ecosystems” (Muñoz & Branzei, 2021, p. 510). Regenerative value is created through co-creative partnerships of various stakeholders with nature that sustain the life-supporting conditions of surrounding social-ecological systems (Konietzko et al., 2023; Robinson & Cole, 2015).

Despite growing interest in considering nature as a stakeholder and related stakeholder engagement for regenerative value creation (Heikkinen et al., 2019; Kujala et al., 2019, 2022), empirical evidence about it remains limited. Heikkinen et al. (2019) and Kujala et al. (2019) introduced nature-inclusive stakeholder engagement as comprising a wide range of organization-relevant relationships and interactions with, and the roles, needs, and preferences of, nature stakeholders, such as species, rivers, forests, or mineral resources, in value creation. Stakeholder scholars who argue for recognizing nature as a stakeholder have called for further research on such relationships (Oberholzer & Sachs, 2023; Tallberg et al., 2022, 2024). In alignment, scholars suggest addressing non-Western and ecocentric views, such as those grounded in Indigenous epistemological and ontological framings, to more comprehensively embrace businesses’ natural reality (Kortetmäki et al., 2023; Rahman et al., 2024; Sama et al., 2004). Building on these calls, this article asks: *How do Indigenous businesses in the Sierra Nevada consider nature relationships in regenerative value creation, and how do these insights contribute to reconceptualizing nature-inclusive stakeholder engagement?*

To answer this question, I follow a decolonizing research approach that grounds discussions in Two-Eyed Seeing (Bartlett et al., 2012). In this study, Two-Eyed Seeing engages the Indigenous perspectives and lived experiences of the Sierra Nevada on regenerative value creation in dialogue with Western-centered perspectives and managerial conceptualizations of nature-inclusive stakeholder engagement. Thereby, the Indigenous eye maximally embraces the shared cosmovision and ontology of the Indigenous peoples of the Sierra Nevada, while the Western eye is informed by stakeholder research on nature. This explorative, qualitative study draws on multiple sources, including 27 interviews with Indigenous business representatives in the Sierra Nevada, 17 pieces of secondary literature with (co-)authorship from Indigenous peoples of the Sierra Nevada, and field-based observation. Data analysis follows the primarily inductive and explorative Gioia methodology (Gioia et al., 2013).

This article theoretically advances and reconceptualizes nature-inclusive stakeholder engagement (Heikkinen et al., 2019; Kujala et al., 2019) in three ways. First, I examine how nature relationships are considered in a place-based Indigenous business context, deepening the understanding of stakeholder engagement for regenerative value creation (Das & Bocken, 2024; Muñoz & Branzei, 2021). The studied Indigenous businesses of the Sierra Nevada offer insights into how multidimensional regenerative value can emerge from a nature-inclusive stakeholder engagement mindset and related interactions. Second, based on these empirical insights provided by the Indigenous eye and put into dialogue with the Western eye, I discuss the multidimensional nature-inclusive stakeholder engagement. The latter emphasizes a nature-sensitive organizational mindset, embraced by organization-internal, nature-connected human proxies, that informs physical, emotional, mental, and spiritual nature relationships. Third, I show how adopting a decolonizing research approach can advance nature-inclusive stakeholder engagement research. This approach emphasizes the importance of respectfully engaging with the millennia-old cosmovisions, knowledges, and experiences of Indigenous peoples, as such engagement can contribute to further challenging anthropocentric and Western biases in business research and practice that impede sustainable pathways (Bastien et al., 2023; Love, 2020).

The remainder of this article is structured as follows. I first provide the theoretical background by reviewing Western-centered research on nature-inclusive stakeholder engagement before introducing the cosmovision and ontology that inform the nature relationships of Indigenous businesses in the Sierra Nevada. Following Two-Eyed Seeing, I discuss the basic assumptions of nature-inclusive stakeholder engagement. I then introduce the empirical setting and reflect on the research methodology, including the decolonizing research approach. Next, grounded in the analogy of weaving, I present the empirical results on the nature-inclusive stakeholder engagement mindset, interactions, and related regenerative value explored in the Sierra Nevada. I conclude by discussing the article’s findings, implications for management and organizational research and practice, limitations, and areas for further study.

## Theoretical Background

### The Western Perspective on Nature-Inclusive Stakeholder Engagement

Since the mid-1990s, several stakeholder scholars have argued for considering nature as a stakeholder to help address socio-ecological challenges and potentially yield regenerative value (Haigh & Griffiths, 2009; Hart & Sharma, 2004; Starik, 1995). These scholars call for integrating nature and its constituents as “the primary and primordial stakeholder[s] of the firm” (Driscoll & Starik, 2004, p. 55), with the latter even becoming non-human “partners” (Kortetmäki et al., 2023, p. 27). Doing so may enable businesses to “create spiritual, cultural, social, environmental and economic well-being” through relational approaches that create regenerative value, as seen in Māori businesses (Spiller et al., 2011, p. 153). However, much of the research on nature as a stakeholder remains embedded in conceptual-theoretical debates, like posthumanism (Sayers et al., 2022) or (eco)feminist organizational research (Calás & Smircich, 2023), which emphasize relationships with nature for sustainable value creation.

Heikkinen et al. (2019) and Kujala et al. (2019) introduced the concept of nature-inclusive stakeholder engagement, referring to the integration of relationships and interactions between human and nature stakeholders into organizations’ activities, such as in value-creation practices. “Nature stakeholders” refers to living entities in relationship with other stakeholders, including ecosystems such as rivers, forests, and coral reefs, or species thereof, such as animals and plants, that are directly or indirectly affected by or affect an organization’s activities (Kortetmäki et al., 2023). In addition, scholars suggest including “non-living” nature stakeholders, such as water, rocks, or mineral resources, in stakeholder engagement (Gulari et al., 2025; Kujala et al., 2019; Waddock, 2011). Consequently, organizations can concretize their direct interactions and relationships with heterogeneous nature stakeholders, which may be mutually beneficial or harmful (Driscoll & Starik, 2004; Sama et al., 2004).

Stakeholder research that incorporates nature implicitly reveals three basic assumptions about nature-inclusive stakeholder engagement. First, such research recognizes specific nature entities relevant to an organization’s operations as legitimate stakeholders with their own voice, power, and integrity (Kortetmäki et al., 2023; Kujala et al., 2019). Instead of the distant representation of nature through third-party human proxies, such as environmental interest groups in traditional stakeholder research (Freeman, 2010; Freeman et al., 2010, 2017), nature-inclusive stakeholder engagement embraces a more direct, organization-internal representation of heterogeneous nature stakeholders (Clifton & Amran, 2011; Heikkinen et al., 2019). This emphasis on heterogeneous stakeholder relationships highlights the underlying relational stakeholder view, which embraces stakeholder engagement as a relational and collaborative process rather than a transactional one (Kujala et al., 2022; Post et al., 2002).

Second, nature-inclusive stakeholder engagement aligns with the ecocentric paradigm and scholars’ call to foster ecocentric stakeholder engagement (Gulari et al., 2025; Kortetmäki et al., 2023; Tallberg et al., 2022). Ecocentrism, unlike anthropocentrism, ascribes intrinsic value to nature “independent of human values and human consciousness” (Gladwin et al., 1995, p. 886). Consequently, organizations have a moral duty to ensure the well-being of nature stakeholders by considering nature as the enabler of life (Purser et al., 1995) and following the principles of sharing and caring when interacting with nature (Waddock, 2011).

Third, nature-inclusive stakeholder engagement may yield regenerative value by integrating nature and human stakeholders in joint value-creation processes (Robinson & Cole, 2015; Spiller et al., 2011). Stakeholder relationships for regenerative value creation may restore (i.e., compensate for negative impacts), preserve (i.e., avoid negative or create net-zero impacts), and/or enhance societal well-being and planetary health (i.e., create net-positive impacts) (Hahn & Tampe, 2021). In comparison with stakeholder value, which can be economic, social, and/or environmental (Billiet et al., 2023; Freudenreich et al., 2020), regenerative value is grounded in ecocentrism and embraces humans as an inseparable and integral part of nature who co-create value with and for nature (Muñoz & Branzei, 2021).

To advance the inclusion of nature in organizational stakeholder relationships, scholars call for embracing Indigenous epistemologies and ontologies, given Indigenous peoples’ extensive knowledges of and profound relations with nature (Sama et al., 2004; Tallberg et al., 2022; Waddock, 2011). One such well-conserved epistemology and ontology is the place-based experiences and shared cosmovision of the Indigenous peoples of the Sierra Nevada.

### Nature Relationships and Cosmovision of the Indigenous Peoples of the Sierra Nevada

Altmann (2020) urges aiming for intercultural translation when weaving territory-bound Indigenous epistemologies and knowledges into research instead of Western abstraction and decontextualization that omits the concrete at the cost of “epistemic extractivism” (p. 93). Here, I make a modest—and by no means complete—attempt to engage in the intercultural translation of the basic assumptions of the holistic epistemology and place-based, *living* cosmovision (*cosmovisión*) shared by the Arhuaco, Kogui, Wiwa, and Kankuamo peoples. Literary contributions by these peoples illustrate the core pillars and role of relationships with nature in their cosmovision, which informs their way of life, including business activities.

The cosmovision or worldview of the four Indigenous peoples of the Sierra Nevada underlies basic assumptions grounded in relationality and reciprocity while emphasizing duality (Mora Calderón & Villafaña Chaparro, 2018; Sauna et al., 2012). Duality concerns complementarities in the universe, such as cold and hot, male and female, or day and night, while human beings are understood as an integral part and kin of nature (Mestre, 2007). At the core of this cosmovision, rooted in the sacred territory of the Sierra Nevada, lies an ecological ethic that aims to foster a spiritually harmonious coexistence among all beings in the universe (Izquierdo Mejía, 2021; Pinto-Marroquin et al., 2022). To the Indigenous peoples of the Sierra Nevada, everything existing physically in nature, moving or non-moving, is interrelated with and complemented by a spiritual counterpart (i.e., a spiritual father or spiritual mother) which defines the origins, time, organization, space, and purpose of the material being (Mestre, 2007). Via spiritual rituals that reward Mother Nature, known as payments (*pagamentos* or *labor tradicional*) and conducted at interconnected sacred spaces in nature (e.g., specific hills, lakes, or stones), the Indigenous peoples of the Sierra Nevada restore and maintain the energetic flows, order, balance, and spiritual communication among all beings in their territory (Pinto-Marroquin et al., 2022; Torres Izquierdo, 2021).

The Arhuaco, Kogui, Wiwa, and Kankuamo peoples understand their territory as a living being rather than a geographically defined physical space or property, representing a human body that consists of material and spiritual beings (Beltrán Armenta, 2017; Izquierdo Mejía, 2021). The permanent flow of relationships among these beings gives meaning to the territory, which constitutes the origin and “Heart of the World” (*El Corazón del Mundo*) (Mestre, 2007). The territory provides the “code” for the orally delivered, millennia-old norms (Law of Origin) that define the identity, culture, spirituality, knowledge base, law, governance systems, and natural order of these Indigenous peoples, guaranteeing the permanence of life (Mestre, 2007; Sauna et al., 2012). Moreover, the Law of Origin (*Ley de Origen*) defines the nature of the interaction of humans with other nature beings, including the use and management of the territory and its components, oriented toward maintaining harmony within the territory, with Mother Nature, and all beings to ensure universal well-being (Beltrán Armenta, 2017). Izquierdo Mejía (2021), a self-identified Arhuaco, defines this harmony as “a balance between the self, the (natural) environment, the forces of nature, the worldview, good living (*buen vivir*), and living together in community,” involving maintaining the living space “where different forms of solidarity and respect for nature materialize” (p. 46, own translation).

Finally, the principles of relationality and reciprocity inform dynamic relationships between the unit and the whole, unfolding within a system of networks comprising spiritual, mental, emotional, and physical relationships among coexistent beings (Beltrán Armenta, 2017). Within this system, every action constitutes a collective, relational, and reciprocal act between humans, other nature beings, and nature as a whole (Torres Izquierdo, 2021). According to the Indigenous peoples of the Sierra Nevada, disrespect for and harm caused to nature beings not only violates the Law of Origin but also affects “the maintenance of life, health, and well-being on the planet” (Pinto-Marroquin et al., 2022, p. 4). Consequently, every action needs to be undertaken with minimal adverse effect on all other beings, ascribing to the Indigenous peoples of the Sierra Nevada the roles of conscious guardians and caretakers of the territory and Mother Nature (Izquierdo Mejía, 2021). While these principles and the basic assumptions of the cosmovision of these peoples inform their consideration of (kinship) relationships with nature (beings) in regenerative economic activity, related literature is scarce.

### Reconceptualizing Nature-Inclusive Stakeholder Engagement through Two-Eyed Seeing

Nature-inclusive stakeholder engagement as a Western construct grounded in stakeholder theory differs in its underlying epistemology and ontology from that embraced by the Indigenous peoples of the Sierra Nevada. How can these different paradigms be leveraged to reconceptualize nature-inclusive stakeholder engagement?

Two-Eyed Seeing (*Etuaptmumk*), a decolonizing guiding principle, analytical lens, or ethical protocol for research, promotes co-learning from and weaving back and forth between distinct and whole knowledge systems through a non-confrontational approach (Bartlett et al., 2012; Rankin et al., 2023). Bartlett et al. (2012) define Two-Eyed Seeing as refer[ing] to see[ing] from one eye with the strengths of Indigenous knowledges and ways of knowing, and from the other eye with the strengths of Western knowledges and ways of knowing, and to using both these eyes together, for the benefit of all (p. 335). Rooted in the Mi’kmaq traditions of North America and developed by Elder Dr. Albert Marshall, Two-Eyed Seeing reflects overarching concepts associated with distinct Indigenous knowledges, such as centering interconnectedness and relating elements to the whole (Rankin et al., 2023). This lens has been widely adopted, including in organizational research (Arjaliès & Banerjee, 2024; Colbourne et al., 2020), to promote a more equal dialogue between Indigenous and Western knowledges (Broadhead & Howard, 2021; Wright et al., 2019). However, Two-Eyed Seeing must not be mistaken for appending pieces of Indigenous to Western knowledges, nor merging two distinct knowledge systems into one (Rankin et al., 2023). Instead, this lens fosters complementing, respecting, and enriching distinct wisdoms (Arjaliès & Banerjee, 2024).

In this article, I adopt Two-Eyed Seeing as an analytical lens to reflect how the consideration of nature relationships—through lived experiences, based on their cosmovision, and anchored in their interests—of Indigenous businesses of the Sierra Nevada (the Indigenous eye offered by the Arhuaco, Kogui, Wiwa, and Kankuamo peoples) can guide nature-inclusive stakeholder engagement (the Western eye) toward new imaginaries. This approach facilitates a dialogue that recognizes that “the ontological limits of Western scholarship” (Arjaliès & Banerjee, 2024, p. 5) regarding analytically driven stakeholder engagement prevent fully embracing the holistic and different ways of knowing (incl. experiential knowing) and interacting with nature by the Indigenous peoples of the Sierra Nevada. On the other hand, Western scholarship on nature-inclusive stakeholder engagement explains how organization-relevant nature relationships can be systematically integrated into value creation approaches (Heikkinen et al., 2019; Kujala et al., 2019). By engaging in this dialogue, the Indigenous epistemology and ontology of the Sierra Nevada can inform a reconceptualization of the consideration of relations between humans and nature beings that have a “stake” in business activity (i.e., nature stakeholders) toward a more comprehensive regenerative approach.

The Indigenous eye and the Western eye inform this dialogue as follows: The Indigenous eye in this article perceives using a relational ontology that centers on creating, valuing, and sustaining harmonious, reciprocal, and profound human-nature relationships in economic activity, wherein nature acts as a living being and kin central to universal well-being (Fan, 2024; Torres Izquierdo, 2021). The Western eye of nature-inclusive stakeholder engagement perceives through the relational stakeholder view (Kujala et al., 2022; Post et al., 2002) and provides an analytical-managerial approach to integrating the roles, needs, and preferences of nature stakeholders in organizational activities (Heikkinen et al., 2019; Kujala et al., 2019). Table 1 summarizes the basic assumptions of nature-inclusive stakeholder engagement as perceived from the Western eye and the Indigenous eye of the Sierra Nevada.

**Table 1.** Basic Assumptions of Nature-Inclusive Stakeholder Engagement Through the Analytical Lens of Two-Eyed-Seeing.

Key dimensions	Western eye (of nature-inclusive stakeholder engagement)	Indigenous eye (of the Sierra Nevada)
Relational consideration of nature	*Analytical* approach of considering nature stakeholders as *particularized entities* from a relational stakeholder perspective	*Experiential* approach of considering nature *beings* from a relational and reciprocal perspective, emphasizing dynamic relations between the *unit and the whole*
Nature-related embeddedness	*Ecocentric perspective* ascribing intrinsic value to nature stakeholders and moral duties to organizations, fostering *sharing and caring attitudes* toward nature stakeholders	Ecological ethic promoting *spiritually harmonious coexistence* between and *respectful interactions* with nature beings by ascribing Indigenous communities as *guardians and caretakers* of Mother Nature
Regenerative value creation	Value co-creation between human and nature stakeholders, yielding *societal well-being and planetary health* by understanding *humans as an integral part of nature*	Value co-creation between humans and nature beings, which are understood as *kins*, yielding *universal well-being* by maintaining *harmony* within the territory and among nature beings

## Empirical Setting

The business activities of the Arhuaco, Kogui, Wiwa, and Kankuamo peoples, informed by their largely preserved ancestral knowledge and culture, provide valuable insights into the consideration of nature relationships for regenerative value creation. These four Indigenous peoples inhabit the Sierra Nevada and are among the 115 Indigenous ethnicities in Colombia, where over 65 Indigenous languages are spoken (Departamento Administrativo Nacional de Estadística [DANE], 2019). The Sierra Nevada, one of the world’s most biodiverse ecosystems, is located on the Colombian Caribbean coast in the departments of Magdalena, César, and La Guajira (United Nations Educational Scientific and Cultural Organization, 2023), and is home to over 85,000 people self-identifying as Arhuaco, Kogui, Wiwa, and Kankuamo (DANE, 2021). These people are the descendants of up to 1 million Tayrona people who inhabited their ancestral territory in pre-Hispanic times for over three millennia before being almost wiped out during Spanish colonization (Niño Izquierdo, 2017).

The four Indigenous peoples of the Sierra Nevada share a common worldview or cosmovision (see theoretical background), cultural base, and ancestral territory (Beltrán Armenta, 2017). The latter is physically demarcated by the Black Line (*Línea Negra*), defined by sacred sites located around a 23,435 square-kilometer sacred terrestrial and maritime area, with the Sierra Nevada mountain range at its center (Izquierdo Mejía, 2021). Despite the Colombian government’s recognition and legal protection of the territory as a sacred site since 1973 (e.g., Decree 1500 of 2018 from the Colombian Ministry of the Interior), the territory has been affected by ongoing neo-colonial pressures (Izquierdo & Viaene, 2018). Territorial expropriation and exploitation, forced displacement, violence, oppression, social exclusion, and forced assimilation have seriously affected the Indigenous peoples of the Sierra Nevada, including their commercial activity (Giraldo Salazar et al., 2021b; Izquierdo Mejía, 2021).

Throughout their history, the most dominant form of commercial activity among the Indigenous peoples of the Sierra Nevada has been community-based bartering (*trueque*), primarily involving agricultural goods (Beltrán Armenta, 2017; Torres Izquierdo, 2021). More recently, these Indigenous communities have engaged in commercial activities beyond self-sustenance, earning money while upholding their traditional values and principles (Giraldo Salazar et al., 2021a). This capitalist commercial activity is a coping mechanism intended to ensure their physical (e.g., covering basic needs) and cultural survival (e.g., regenerating sacred spaces), resist neo-colonial oppression, and prevent further discrimination and exclusion by parts of the majoritarian Colombian society (Torres Izquierdo, 2021; Zapata Izquierdo, 2020).

Most of the small- and medium-sized, community-based Indigenous businesses in the Sierra Nevada operate in organic agroforestry and agriculture (mainly coffee, cacao, avocado, and other fruit cultivation, and panela and honey production), tourism (e.g., ancestral tours and retreats, accommodation, and gastronomy), and traditional handicrafts (e.g., *mochila* bags) (Giraldo Salazar et al., 2021b). Informed by the Law of Origin, these businesses take account of their territory’s environmental, social, economic, cultural, and spiritual needs and interests.

## Methods

### Conducting Indigenous Research as a Non-Indigenous Scholar

As a non-Indigenous scholar, I adopted a decolonizing research approach to ensure compliance with Indigenous research ethics and maintain ethical responsibility when drawing on the experiences of people oppressed and marginalized by (neo)colonial practices (Banerjee, 2022; Weston & Imas, 2018). Thambinathan and Kinsella (2021) define decolonizing research as “centering concerns and world views of non-Western individuals, and respectfully knowing and understanding theory and research from previously ‘Other(ed)’ perspectives” (p. 1). In organizational research, this approach includes embracing non-Western forms of organizing based on “communal, cooperative, and nature-reverent norms” (Hamann et al., 2020, p. 4).

I developed a five-point approach to guide respectful engagement with Indigenous knowledges and participants, following established methodological principles for Indigenous research (Chilisa, 2012; Love, 2020; Schneider & Kayseas, 2018; Peredo, 2023). The approach involved: (a) adopting a pluriverse perspective (Banerjee, 2022); (b) designing the research to be appreciative, participative, and flexible (Schneider & Kayseas, 2018); (c) immersion in the place-based Indigenous setting (Hamann et al., 2020); (d) maintaining critical reflexivity to minimize Western bias (Weston & Imas, 2018); and (e) preserving the integrity of the Indigenous setting throughout (Alcadipani et al., 2012). For example, I kept a research journal and audio recorded memos to critically track and address blind spots related to my own Western bias.

### Data Collection

**Figure 1.**
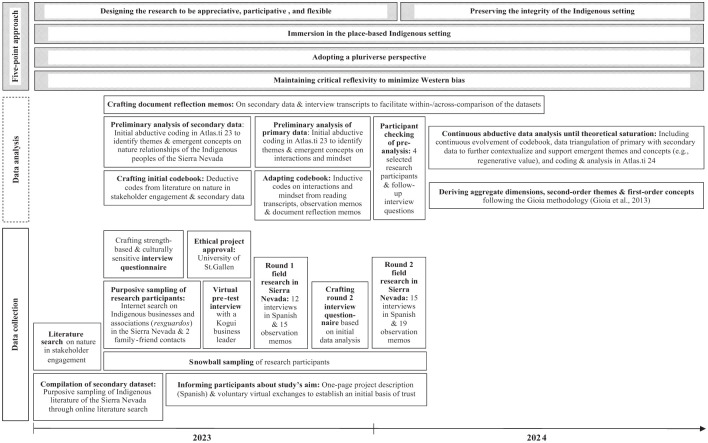
Timeline of Methodological Approach.

This article draws on data from semi-structured interviews, observation, and secondary literature, as outlined in Table 2. Figure 1 shows the timeline of my iterative data collection and analysis process, which drew on grounded theory (Charmaz, 2006).

**Table 2.** Data Description.

Collected data types and dates	Amount in analyzed data format	Use in data analysis
Primary data (Total fieldwork: 4 months)		
*Interviews*		
27 semi-structured interviews with Indigenous business representatives from the Sierra Nevada, lasting between 22 and 128 min (59 min on average) (August–September 2023: *n* = 12; January–February 2024: *n* = 15)	470 pages of text (verbatim transcriptions from audio recordings; A4, font: Times New Roman, 12, 1.5 line spacing)	Insight into the mindset and interactions of organizational representatives of Indigenous businesses in the Sierra Nevada with nature beings and resulting regenerative value (coded during the data analysis process)
*Observation*		
34 observation memos from field research in the Sierra Nevada, lasting between 2 min and 12 min (August–September 2023: *n* = 15; January–February 2024: *n* = 19)	157 min of audio recording (used as summarized memo notes highlighting key insights)	Identification of factors observed during field research that may influence/demonstrate the materialization of the studied mindset, interactions with nature beings, and related regenerative value (identification of emerging themes/concepts during data collection; sensemaking during data analysis)
Secondary data		
Literature (marked with an “*” in the references) 17 pieces of literature (1997–2022): journal articles (*n* = 4), books (*n* = 3), book chapters (*n* = 1), university theses (*n* = 5), booklets (*n* = 4) • fully authored (*n* = 8), majorly co-authored (*n* = 3), and partly co-authored (*n* = 6) by members of the Indigenous peoples of the Sierra Nevada	935 pages of text (in original published format)	Contextualize primary data and insight into the cosmovision/worldview, ontology, culture, socio-economic context, and commercial activity of the Indigenous peoples of the Sierra Nevada that influence the identified mindset, interactions with nature beings, and related regenerative value types (coded during the data analysis process)

To select research participants, I relied on purposive sampling followed by snowball sampling (Cassell, 2015). The participant selection criteria included: (a) physical engagement in an Indigenous business in the Sierra Nevada; (b) self-identification as Arhuaco, Kogui, Wiwa, or Kankuamo by the owners and leaders of these businesses; and (c) the possibility of conducting the interviews in Spanish and in person in the Sierra Nevada. To ensure the greater transferability of findings, I selected participants independent of the affiliated businesses’ industry or size. I started several snowballs, identified through two family-friend contacts and an online search of Indigenous businesses and associations (*resguardos*) in the Sierra Nevada.

As shown in Table 3, I conducted 27 semi-structured interviews in Spanish with 20 Indigenous business leaders and employees from 8 Indigenous businesses in the agriculture, tourism, artisanal, and audiovisual sectors, which are run by 1 or several communities of the 4 Indigenous peoples of the Sierra Nevada. I conducted two rounds of field research in the Sierra Nevada from August to September 2023 and January to February 2024. During the first round, after obtaining ethical project approval from my university and conducting a virtual pre-test with a Kogui business leader, I made 15 observation memos and conducted 12 interviews based on a semi-structured interview guide (see Appendix 1 for the leading interview questions). The latter was informed by an extensive literature search on nature in stakeholder engagement that created this study’s starting point for co-learning promoted by Two-Eyed Seeing, rather than a fixed theoretical framework. During the second round, I made 19 observation memos and conducted 15 interviews, of which 8 included 8 participants I had already interviewed during the first round, and 7 interviews with 6 new participants. Interviewing participants twice helped build trust and deepen emergent concepts from the first round, as well as identify new concepts. Including new participants minimized participant-related biases concerning the first round. The audio-recorded observation memos helped me make sense of the Indigenous experiences and identify emergent themes and concepts. I stopped collecting primary data when no new themes emerged from the data analysis, that is, when theoretical saturation was achieved, and I felt confident that I had grasped the studied phenomenon (Glaser & Strauss, 1967; Howard-Grenville et al., 2021).

**Table 3.** Participant Table.

**Aggregated sample**								
Interviews	Participants	Role	Business & industry^a^	Ethnicity^b^	Gender	Interview modality^c^	Duration	Date
*n* = 27	*n* = 20	Business leader: *n* = 12, employee: *n* = 8	Businesses: *n* = 8, agriculture: *n* = 5, tourism: *n* = 2, artisanal: *n* = 2, audiovisual: *n* = 1	Arhuaco: *n* = 10, Kankuamo: *n* = 5, Kogui: *n* = 3, Wiwa: *n* = 1, non-Indigenous^d^: *n* = 1	Male: *n* = 14, female: *n* = 6	In person: *n* = 19, virtual: *n* = 8, individual: *n* = 22, group (2–3 participants): *n* = 5	26:46h	Round 1: August-September 2023, round 2: January-February 2024
**Round 1**								
Interview code	Participant(s)	Role	Business & industry	Ethnicity	Gender	Interview modality	Duration (HH:MM)	Date (YYYY/MM/DD)
I1	A	Business leader	I (agriculture)	Arhuaco	Male	In person	00:43	2023/8/12
I2	B, C, D	Business leader (B, C), employee (D)	II (agriculture, tourism, artisanal)	Kogui (B, C, D)	Male (B, C), female (D)	In person	01:26	2023/8/28
I3	E, F	Business leader (E), employee (F)	III (agriculture)	Arhuaco (E), non-Indigenous (F)	Male (E, F)	In person	01:23	2023/8/29
I4	G, H	Business leader (G), employee (H)	III (H) / IV (G) (agriculture)	Arhuaco (G, H)	Male (G, H),	In person	01:16	2023/8/30
I5	I, J	Employee (I, J)	III (agriculture)	Kankuamo (I, J)	Female (I), male (J)	In person	02:08	2023/8/30
I6	H	Employee	III (agriculture)	Arhuaco	Male	In person	00:47	2023/8/31
I7	K	Business leader	V (agriculture)	Arhuaco	Male	In person	00:46	2023/8/31
I8	L	Business leader	III (agriculture)	Arhuaco	Female	In person	01:09	2023/9/1
I9	E	Business leader	III (agriculture)	Arhuaco	Male	In person	00:33	2023/9/1
I10	A	Business leader	I (agriculture)	Arhuaco	Male	Virtual	00:46	2023/9/19
I11	M	Employee	III (agriculture)	Kankuamo	Female	Virtual	00:49	2023/9/20
I12	N	Employee	III (agriculture)	Kankuamo	Male	Virtual	00:55	2023/9/20
**Round 2**								
Interview code	Participant(s)	Role	Business & industry	Ethnicity	Gender	Interview modality	Duration (HH:MM)	Date (YYYY/MM/DD)
I13	O	Business leader	VI (audiovisual)	Arhuaco	Male	In person	00:50	2024/1/16
I14	P, Q	Business leader (P, Q)	VII (artisanal)	Arhuaco (P, Q)	Female (P, Q)	In person	00:46	2024/1/16
I15	L	Business leader	III (agriculture)	Arhuaco	Female	In person	01:06	2024/1/17
I16	R	Business leader	III (agriculture)	Arhuaco	Male	In person	00:57	2024/1/17
I17	E	Business leader	III (agriculture)	Arhuaco	Male	In person	00:56	2024/1/17
I18	M	Employee	III (agriculture)	Kankuamo	Female	In person	00:54	2024/1/18
I19	S	Employee	III (agriculture)	Kankuamo	Male	In person	01:09	2024/1/18
I20	N	Employee	III (agriculture)	Kankuamo	Male	Virtual	00:52	2024/1/22
I21	I	Employee	III (agriculture)	Kankuamo	Female	Virtual	00:38	2024/1/22
I22	F	Employee	III (agriculture)	non-Indigenous	Male	In person	01:34	2024/1/22
I23	P	Business leader	VII (artisanal)	Arhuaco	Female	Virtual	00:36	2024/1/23
I24	T	Business leader	VIII (tourism)	Wiwa	Male	In person	01:01	2024/1/24
I25	T	Business leader	VIII (tourism)	Wiwa	Male	Virtual	01:32	2024/1/26
I26	A	Business leader	I (agriculture)	Arhuaco	Male	Virtual	00:52	2024/1/30
I27	B	Business leader	II (agriculture, tourism, artisanal)	Kogui	Male	In person	00:22	2024/2/2

a In total, the interview sample includes eight Indigenous businesses of the Sierra Nevada. One of these businesses was simultaneously active in the agriculture, artisanal, and tourism industries.

b Although the Arhuaco peoples are the largest Indigenous ethnic group in the Sierra Nevada (DANE, 2021), the distribution of the participants’ Indigenous ethnicities in this article is not representative of the latter’s population sizes. The four Indigenous peoples I studied share the same cosmovision and many similarities in their customs and socio-economic contexts. Hence, I prioritized data quality and access to comparable Indigenous businesses over the proportional representation of the four Indigenous ethnicities in my sample.

c I conducted interviews both in person in the Sierra Nevada and via video calls during or after field research due to unpredictable changes in the research schedule. However, online interviews were only conducted with research participants I had met in person before in the Sierra Nevada to ensure an equal sense of trust and familiarity during all interviews. Accommodating research participants’ preferences while adhering to my decolonizing research approach, I conducted individual and group interviews.

d Although all participants self-identified with one of the four Indigenous peoples of the Sierra Nevada, one participant identified as non-Indigenous. However, this participant had been employed by an Indigenous business in the Sierra Nevada under Indigenous leadership for several decades and demonstrated a profound knowledge of the former’s ancestral culture and cosmovision. The information from this participant was included in the data analysis and limited to information on nature relationships by the Indigenous business (representatives) he had worked with, excluding his own nature relationships.

I purposefully sampled secondary data (see * in the references) through an online literature search based on three selection criteria: (a) involvement of Indigenous authorship from the Sierra Nevada, (b) discussion of the Indigenous peoples of the Sierra Nevada’s cosmovision, culture, socio-economic context, or commercial activity, and (c) digitally available literature in Spanish or English. The (co-)authorship by the Indigenous peoples of the Sierra Nevada of the 17 retrieved literary works minimized additional layers of interpretation in the data analysis.

### Data Analysis

The data analysis process primarily followed the inductive and exploratory Gioia methodology (Gioia et al., 2013) to ground the derived concepts and themes solidly in the data and thus in the ontology and epistemology of the Indigenous peoples of the Sierra Nevada. For a preliminary abductive analysis and coding of the secondary data in Atlas.ti 23, an initial codebook was developed. I derived deductive codes on nature-inclusive stakeholder engagement interactions (e.g., communicating with or learning from nature beings/ stakeholders) from the literature review on nature in stakeholder engagement. During and after conducting the first round of field research, I continuously adapted the codebook while creating inductive code(s) (categories) that emerged from a preliminary analysis of my primary data in Atlas.ti 23, including a (re-)reading of and writing of document reflection memos on interview transcripts and secondary data, taking notes on the observation memos, and an initial coding round. Document reflection memos highlighted key constructs and emerging themes, facilitating within- and cross-comparison of primary and secondary data. The inductive codes extended the deductive codes on interactions with nature beings by the studied Indigenous businesses and gave rise to new code categories for the related mindset (e.g., consciousness or relational thinking). For the inductively derived mindset, I coded the principles, values, and perceptions of the research participants that influenced their attitudes toward, sensemaking of, and interaction with nature beings in their business activities (Hahn et al., 2014; Meath et al., 2024). This initial data analysis round created the basis for the interview guide used in the second round of field research, which further unpacked key themes, categories, and emergent concepts (e.g., the type of value created, spirituality in mindset, or prioritizing nature beings).

To ensure data validity, I relied on interpretative participant checking (Lindheim, 2022). I discussed my findings from the pre-analysis of the first round in person with four Indigenous research participants at the beginning of the second round of field research, showing them a visualization of the identified nature-inclusive stakeholder engagement mindset and “mechanisms” (later theorized as “interactions” to better reflect the experiential approach of the Indigenous eye). In addition, during the second round of field research, I introduced follow-up questions that deepened my preliminary theorization after asking the leading interview questions. This approach is built on research that shows how participant checking helps prevent the romanticization, exoticization, idealization, and reductionism of Indigenous experiences by fostering an accurate portrayal of the concepts derived from Indigenous knowledges and experiences (Alcadipani et al., 2012; Weston & Imas, 2018).

**Figure 2.**
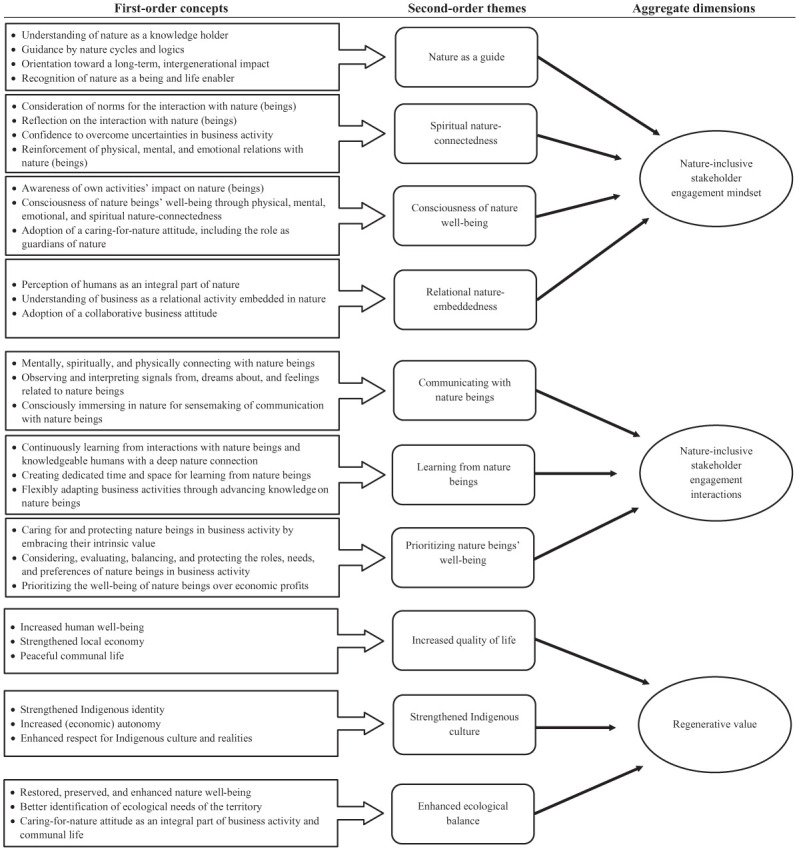
Data Structure.

I more comprehensively coded my entire dataset abductively and analyzed the data in Atlas.ti 24 after finishing data collection until I reached theoretical saturation. Once I had gained a better understanding of the emerging concepts and themes from my primary data, I solidified the related first-order concepts and second-order themes by triangulating them with the coded secondary data (Gioia et al., 2013). Figure 2 illustrates the resultant data structure, which includes the aggregate dimensions conceptualized based on the second-order themes and on focusing on the research question. I derived the mindset and regenerative value dimensions inductively based on the Indigenous eye of the Sierra Nevada and the interactions dimension abductively and partly informed by the Western eye.

## Findings

To present the findings on the nature-inclusive stakeholder engagement mindset, related interactions, and the resulting regenerative value seen from the Indigenous eye of the Sierra Nevada, I employ a literary analogy of weaving. The weaving or knitting (*tejer*) of traditional fique, cotton, and wool bags (*mochilas*) is a millennia-old tradition and spiritual practice, deeply entrenched in the cosmovision, culture, and everyday lives of Indigenous women of the Sierra Nevada (Rodríguez-Burgos et al., 2014; Rodríguez Cifuentes et al., 2020a). Although this section relies on the weaving analogy, the findings are representative of the Indigenous businesses studied across different industries.

### Nature-Inclusive Stakeholder Engagement Mindset: Weaving Principles

The weaving of traditional bags by the four Indigenous peoples of the Sierra Nevada is directly connected to their Law of Origin, which defines norms and principles for all aspects of life, builds collective identity, and is aimed at the care of Mother Nature (Rodríguez-Burgos et al., 2014; Rodríguez Cifuentes et al., 2020a). Similarly, the interactions of the studied Indigenous businesses with nature beings relevant to their business activity (or “nature stakeholders” as seen from the Western eye) underly four interrelated key determinants of what I have identified as a nature-inclusive stakeholder engagement mindset: *nature as a guide*, *spiritual nature-connectedness*, *consciousness of nature well-being*, and *relational nature-embeddedness*. A mindset comprises cognitive frames or mental models that influence the thinking, interpretation, attitudes, and actions of individuals and organizations (Hahn et al., 2014; Meath et al., 2024). The identified mindset, tied to the shared cosmovision of the Arhuaco, Kogui, Wiwa, and Kankuamo peoples, reveals the cognitive frames that influence the studied business representatives’ attitudes toward, sensemaking of, and interaction with nature beings.

#### Nature as a Guide

Conceiving of nature as a guide lies at the core of the identified mindset. This conception assigns nature the attribute of a source of knowledge that the Indigenous business representatives consider in their daily business activity, becoming tangible through observation and physical immersion in nature or consultations with their traditional leaders and authorities (*mamos*) who possess a more profound knowledge of nature (Namen & Villazón Zalabata, 2020). These nature consultations ensure guidance by and alignment of business activity with nature’s cycles, such as seasons or lunar phases, and logics, including systemic interrelatedness and regeneration. Relatedly, the studied Indigenous business representatives embrace a long-term, intergenerational impact orientation that considers the well-being of (future) nature beings, including their territory. This consideration is grounded in the recognition of Mother Nature as a being and enabler of all life, consisting of further living beings (Izquierdo Márquez, 2019), as highlighted in the following interview excerpt:

This long-term vision also leads us to think about the territory as a being [and] how we are contributing. [This vision leads us] to reflect from a [. . .] business point of view [, to] say, well, [. . .] if we are impacting the territory as it should be [, the territory will last] for several generations. (Arhuaco male Indigenous business leader, Interview 16)^1^

The guidance of businesses by nature implies strong flexibility in adapting activities while planning according to processes, rhythms, and the well-being of present and future nature beings. Spirituality helps the studied Indigenous businesses live up to this ambition.

#### Spiritual Nature-Connectedness

Through the Law of Origin, spirituality provides norms and principles to the studied Indigenous businesses that support interacting harmoniously with, caring for, and protecting nature (beings) to guarantee the life, health, and well-being of all beings (Izquierdo Mejía, 2021; Sauna et al., 2012). For instance, collective and individual payment rituals (*pagamentos*) embrace the principle of giving-and-taking, including asking for permission to interact with and pay tribute to nature (beings) for her (their) life-enabling role, and precede business activities that include direct nature interventions (Mestre, 2007; Pinto-Marroquin et al., 2022). Hence, spirituality creates room for reflection on the interaction with nature (beings), facilitating harmonious business activities with nature. An Arhuaco male Indigenous business leader emphasized spirituality’s reflective role as follows:

To maintain this connection [with nature], you have to do these traditional activities, [. . .] the payment rituals. It [this connection] is giving something back spiritually, which for me [. . .] means simply taking the time to reflect on this relationship [. . .]. It’s the opportunity we give ourselves as a society, as a family, as members [of the organization], to sit down and think about the relationship one has [with nature]. (Interview 17)

Furthermore, spiritual relations with nature (beings) increase the confidence of Indigenous business representatives of the Sierra Nevada that they can overcome uncertainties by reinforcing a heightened sense of guidance that goes beyond rational thinking. Overall, a spiritual mindset reinforces physical (e.g., rituals including physical nature interactions), mental (e.g., moral norms and imaginaries concerning nature interactions), and emotional relations with nature (e.g., emotional strength through well-intentioned rituals), enhancing the consciousness and consideration of nature beings’ well-being in business activity.

#### Consciousness of Nature Well-Being

The participants demonstrate a strong awareness of how their activities affect nature as a whole and particular nature beings, including humans, immediately and in the long term. This consciousness guides their constant reflection and learning process. A Kogui male Indigenous business leader explained the underlying rationale:

We humans must be more conscious and start to see where we’re going, where we’re stepping, daily. [. . .] This occurs mentally, in the way we act, also in the way we think, to seek more than anything else [an understanding of] what nature is. Despite any work we do, we must always take nature into account and somehow take care of her [. . .]. (Interview 2)

A “caring-for-nature attitude” (Connolly & Cullen, 2018), rooted in the Law of Origin and the Indigenous cultures of the Sierra Nevada (Niño Izquierdo, 2013; Torres Izquierdo, 2021), guides participants in fulfilling their role as guardians of nature. This attitude ascribes respect and intrinsic value to nature beings and involves a profound consciousness of and concern for their well-being (Ortiz Rodríguez, 1997), enabling the creation of regenerative value. Indigenous business representatives’ deep awareness of the intentions, consequences, and overall impact of their activities on the ecological balance is strengthened through their physical, mental, emotional, and spiritual connection with nature (beings).

#### Relational Nature-Embeddedness

Acknowledging all beings’ coexistence and interconnectedness in nature includes perceiving humans and businesses as part of (rather than in possession of) an existing ecosystem composed of heterogeneous nature beings. Hence, understanding humans as an integral part of nature emphasizes businesses’ responsibility for considering ecological balance through harmonious coexistence with nature beings (Granados Castellanos et al., 2012). The studied businesses understand their activities as embedded in interdependent, reciprocal, and bi-directional relationships with nature and human beings. These relationships can be beneficial or destructive concerning regenerative value creation, depending on an organization’s intentions. In the following, an Arhuaco female Indigenous business leader highlights the relevance of adopting a relational view of business:

I think it’s so important, [to consider] all the relationships with people and also with everything, [. . .] with nature, yes. I think we are part of. . ., we have to see it [life] as if it were an ecosystem, everything is connected. If something [bad] happens, and I know, this fountain, this wetland, etc., it’s going to happen, it’s going to end many lives of animals, etc., and that creates an imbalance. (Interview 15)

The perception of business as a relational activity, dependent on and impacting nature (beings), fosters a collaborative business attitude among the studied Indigenous businesses. This attitude involves the consideration of the diverse needs and interests within the territory, including collaboration with non-Indigenous actors for regenerative value creation, grounded in the understanding that organizational success is interdependent with the well-being of nature.

Combined, the key determinants of the mindset enable a comprehensive consideration of nature beings in business activity. Next, I present the materialization of this mindset by discussing the interactions between the studied Indigenous businesses and nature beings, or, when seen through the Western eye, nature-inclusive stakeholder engagement interactions.

### Nature-Inclusive Stakeholder Engagement Interactions: Weaving the Mochila

The weaving of a *mochila* informed by the Law of Origin requires interacting with nature (beings) on a spiritual, mental, emotional, and physical level (Rodríguez-Burgos et al., 2014). Analogously, I identified nature-inclusive stakeholder engagement interactions, explaining how Indigenous businesses in the Sierra Nevada engage with nature beings, including *communicating with them*, *learning from them*, and *prioritizing their well-being*.

#### Communicating with Nature Beings

Mentally, spiritually, and physically connecting with nature beings helps the studied Indigenous business representatives communicate non-verbally with those beings who guide their activities. By embracing spiritual nature-connectedness, these organization-internal members establish deep connections with the spiritual elements of nature beings (Mestre, 2007; Mora Calderón & Villafaña Chaparro, 2018). These connections, combined with frequently paying attention to and interpreting the signals of nature beings in their environment, support the participants in maintaining business activity in harmony with nature by understanding the territory’s well-being and these beings’ roles, needs, and preferences related to business. Nature beings’ signals can appear at varying frequencies and include sounds, physical changes in the environment (e.g., colors, animal behavior), natural phenomena, feelings, and dreams. Hence, communicating with nature beings implies immersion in nature to observe, read, and interpret their signals, thereby enhancing the understanding of a business’s nature-embeddedness. An Arhuaco male Indigenous business leader stressed the role of communicating with nature beings in his organization, as follows:

Communication, we think of it like in a couple’s relationship. If communication fails, the relationship gets damaged, right? You lose because you no longer understand what’s going on. But nurturing communication allows you to stay connected in some way, yes? [. . .] so that you don’t forget about that being, right? If you don’t communicate, you forget that it [being] exists too. (Interview 17)

Finally, communicating with nature beings enables businesses to better understand and minimize the damage caused to, and by, them through continuous learning.

#### Learning from Nature Beings

The understanding of nature as a guide and knowledge holder implies the relevance of learning from direct interactions with nature beings and knowledgeable humans with a deep nature connection (e.g., *mamos*). This learning helps participants better understand and embrace nature’s logics and cycles in business activities by recognizing how they affect distinct ecosystems and their particular beings. An Arhuaco male Indigenous business leader illustrated how learning from nature permeates his organization:

I believe that this view, which encompasses both relationships and a systems perspective, as observed and learned in nature, can be integrated into organizational processes. [. . .] They are like logics of understanding life—that one can take from seeing how nature relates to an organization, and [. . .] you start to act according to these logics. (Interview 17)

The studied Indigenous businesses perceive learning from nature (beings) as a life-long process that they institutionalize through dedicated time and space in everyday business. For instance, spiritual rituals involving interactions with nature (beings) and profound reflection thereof foster ecological awareness (Pinto-Marroquin et al., 2022). Being conscious of the well-being of nature beings by advancing knowledge about them helps the interviewed Indigenous business representatives avoid and more flexibly adapt activities that cause harm to these beings and strengthen those activities that already have a positive impact. Hence, learning from nature beings informs the studied businesses’ prioritization of the well-being of these beings.

#### Prioritizing Nature Beings’ Well-Being

The analyzed Indigenous businesses in the Sierra Nevada aim to prioritize the well-being of nature beings in their activities by caring for and protecting them, thereby maintaining business in harmony with nature and ensuring long-term regenerative value creation. However, prioritizing nature beings’ well-being based on their intrinsic value rather than their relationship to economic profit excludes a generic prioritization of nature over human beings. As integral parts and beings of nature, the studied businesses perceive humans as equally important for regenerative value creation and consider the satisfaction of basic human needs. Hence, these businesses institutionalize the prioritization of nature beings’ well-being over economic gains in strategies and daily business activities that consider, evaluate, balance, and protect nature beings’ roles, needs, and preferences, along with those of humans. The following quote shows how an Arhuaco female weaver prioritizes nature beings over economic gains in her work:

If we see that our *mochila* or something is against that [nature], we stop doing it because, for us, the most important are nature, the water, the trees, all those [beings]. (Interview 14)

Generally, no prioritization among nature beings occurs within the studied businesses, although they take special care of nature beings that uphold the foundations of life, and that play a vital role in their cosmovision and spiritual world, such as water, soil, air, and mountains.

Combined, the three nature-inclusive stakeholder engagement interactions discussed can contribute to systematically incorporating the roles, needs, and preferences of nature beings into business activities, yielding regenerative value.

### Regenerative Value: Carrying the Fruits

Representing their cultural identity, the *mochilas* accompany the Indigenous peoples of the Sierra Nevada in their everyday lives, carrying documents, personal items, or the harvest of fruits and vegetables (Rodríguez Cifuentes et al., 2020b). Analogously to the *mochila*’s wide-ranging utility, the studied community-based Indigenous businesses contribute to various types of regenerative value. Regenerative value refers to the interconnected social, cultural, and ecological net-positive contributions that organizations and stakeholders make, co-created with nature, to the life-supporting conditions, health, and resilience of, and well-being in, social-ecological systems (Hahn & Tampe, 2021; Robinson & Cole, 2015). I identified three types of regenerative value that can result from nature-inclusive stakeholder engagement: *increased quality of life, strengthened Indigenous culture*, and *enhanced ecological balance*.

#### Increased Quality of Life

The studied Indigenous businesses view their activities as a means of enhancing the quality of life in their territory by helping to satisfy communities’ socio-economic needs. Their deep embeddedness and immersion in their territory help these organizations to understand these needs, which, according to them, can only be fulfilled if nature is intact. For instance, increased human well-being, such as improved health, can result from enhanced food security through the diversification of food sources, achieved by generating additional income locally and adopting strategies that mimic the diversity inherent in nature. An Arhuaco male Indigenous business leader emphasized how food security is enhanced through agroforestry by considering nature beings’ interrelatedness in ecosystems:

We’ve [got] to sustain ourselves, [we’ve got] to live from the cultivation of something, but we’ve [got] to make sure that [we grow] a crop that isn’t so destructive, for example, mangos. If we plant good mangos, then we have shade there [in the cultivation] because there they produce air, they produce food for us. This program of [growing] cocoa [and] coffee, there we’re planting trees; at the same time, it [program] sustains us. (Interview 4)

In addition, the studied Indigenous businesses invest in local infrastructure and education, generating intergenerational labor and income within their territory’s communities. These businesses communally share the financial gains they generate by prioritizing the quality of their products and services while consciously interacting with nature beings. This consideration of nature beings’ (including humans’) needs and preferences allows these businesses to foster a more peaceful communal life based on the pillars of their cosmovision.

#### Strengthened Indigenous Culture

Business activity that upholds their cosmovision and yields a positive impact on their territory fosters the cultural identity of the studied Indigenous business representatives. For instance, buying back expropriated sacred land with earned income has, in some cases, strengthened social cohesion and identity among the Arhuaco, Kogui, Wiwa, and Kankuamo peoples. By engaging in Indigenous-led business, members of these communities protect certain aspects of their identity by deciding which cultural practices and knowledges are upheld and transmitted to non-Indigenous stakeholders and in what ways the ancestral territory gets involved. A Wiwa male Indigenous business leader highlighted the strengthening of Indigenous culture through the enhanced autonomy of his tourism activities:

We created our own system [. . .] from a [Wiwa] origin, from a principle of self-governance and governability in our territory. [. . .] That’s why [. . .] all these [economic] resources also support the development and strengthening of our Indigenous peoples [. . .]. I speak a lot from my point of view of how I protect and how I create a barrier, both legally and commercially, so that there’s a line that we cannot cross. (Interview 24)

Furthermore, the studied businesses enhance the autonomy of their communities, extending beyond the generation of direct income. For instance, these businesses foster youth leadership and women’s roles in Indigenous communities by assigning them leadership positions and supporting their participation in regenerative business. By yielding regenerative value that also benefits non-Indigenous stakeholders, the studied businesses increase their legitimacy and respect for their culture among the majoritarian society. This respect preserves the Indigenous culture that sustains the protection of the territory’s ecological balance.

#### Enhanced Ecological Balance

Integrating the roles, needs, and preferences of nature beings into daily business activities supports the studied Indigenous businesses in restoring, preserving, and enhancing the well-being of nature in their territory. A Kankuamo male Indigenous business employee highlighted this integration in an agroforestry context:

When one looks at the environment of a life project [farming] [. . .], there’s an environment with other living beings, in which there’re some plant species, there’re other animal species, [and] even very small species, microorganisms, that’re part of all the production processes. So, when we integrate that entire environment in the territory into that productive unit, we’re obviously going to benefit [. . .] not only the producer but also nature. (Interview 19)

Nature’s well-being becomes visible to the Indigenous business representatives through enhanced biodiversity, for instance, fostered via intentionally produced food for animals or intact ecosystem services such as a running and clean water supply. Directly considering nature beings’ needs in business activities requires a thorough assessment of the territory’s ecological needs, based on physical immersion in nature, which, in turn, facilitates business activities in harmony with nature. Consequently, a strengthened caring-for-nature attitude is more likely to become an integral part of business activity and, possibly, (non-)Indigenous communal life.

The three types of regenerative value discussed are informed by the nature-inclusive stakeholder engagement mindset and interactions studied in the community-based Indigenous businesses in the Sierra Nevada. These value types are not mutually exclusive and may occur to varying degrees depending on an organization’s intentions and forms of organizing.

## Discussion

This article aims to reconceptualize the nature-inclusive stakeholder engagement construct (Heikkinen et al., 2019; Kujala et al., 2019) based on insights from considering nature relationships in regenerative value creation by Indigenous businesses in the Sierra Nevada. Weaving back and forth between the empirical insights delivered by the Indigenous eye of the Sierra Nevada and the Western eye of nature-inclusive stakeholder engagement, I propose a multidimensional nature-inclusive stakeholder engagement construct that is sensitive to Indigenous and regenerative organizing, grounded in an experiential approach.

### Weaving Toward Multidimensional Nature-Inclusive Stakeholder Engagement

The place-based experiences of the studied Indigenous businesses, rooted in and shaped by their territory, inform a more comprehensive stakeholder approach than the one seen through the Western eye for promoting societal well-being and planetary health, as discussed next.

#### Nature-Sensitive Mindset for Considering Nature Stakeholders

Adopting Two-Eyed seeing helps envision a nature-sensitive organizational mindset based on profound and reflexive relationships with nature that enhances the consideration of possibilities and limitations associated with including nature (beings) as (a) stakeholder(s) and guide(s) in value creation. The Western eye of nature-inclusive stakeholder engagement underlies an ecocentric mindset that can help consider natural constraints and leverage nature-based opportunities in value creation (Heikkinen et al., 2019; Kortetmäki et al., 2023). This mindset understands nature stakeholders as “active participants with their own interest and agency in the process at hand” (Gulari et al., 2025, p. 233). Related research either includes nature as a unified stakeholder (Gauthier, 2018; Haigh & Griffiths, 2009; Laine, 2010) or nature entities as particularized stakeholders (Araujo et al., 2022; Kortetmäki et al., 2023; Kujala et al., 2019). In contrast, the Indigenous eye of the Sierra Nevada emphasizes a simultaneous unified (i.e., engaging with nature as a whole) and particularized stakeholder understanding (i.e., engaging with specific nature beings). This eye advances the understanding of nature (beings), which is (are) directly or indirectly related to business activity, as (a) guide(s) with whom profound relationships of care, consciousness, and interconnection are shared. Research on regenerative organizing highlights nature guidance in economic activity through nature-based interpretations and interactions (Gualandris et al., 2024; Muñoz & Branzei, 2021), which require humans “to engage in purposeful, reflexive efforts” (Albareda & Branzei, 2025, p. 3553).

#### Regenerative Interactions Through Profound Physical, Emotional, Mental, and Spiritual Nature Stakeholder Relationships

Seeing through the Indigenous eye and Western eye advances a relational stakeholder approach that captures the roles, needs, preferences, vulnerabilities, and beneficial or destructive characteristics through regenerative interaction with nature stakeholders. The Western eye of nature-inclusive stakeholder engagement primarily focuses on physical interactions with nature stakeholders (Heikkinen et al., 2019; Kujala et al., 2019). Limited to animals, this research also discusses the emotional dimension of nature stakeholder relationships (Connolly & Cullen, 2018; Merskin, 2021; Tallberg et al., 2022, 2024). The insights from the Sierra Nevada show how compassionate, nature-connected, organization-internal individuals who are immersed in their organization’s physical environment (Hart & Sharma, 2004; Merskin, 2021) contribute to regenerative value creation by upholding deep relations when interacting with nature stakeholders. These relations are grounded in caring for and protecting nature stakeholders based on their intrinsic value and interdependence with business success. The Indigenous eye of the Sierra Nevada embraces an experiential approach based on physical, emotional (affective), mental (cognitive), and spiritual engagement, including all nature beings. Organizational research shows that emotional relationships can include nature in stakeholder engagement by knowing through emotions, which expands humans’ empathy regarding non-humans and helps notice, sense, and listen to their signals and interpret their needs and “stakes” (Gulari et al., 2025; Tallberg et al., 2022). Mental nature stakeholder relationships can enable “imaginative knowing,” which encompasses imaginative inquiry and speculation about nature stakeholders beyond rational knowing directed toward creating alternative future scenarios (Gulari et al., 2025; Muñoz & Hernandez, 2024). Ultimately, Indigenous organizing research that adopts an intrinsic stakeholder view explains how spiritual relationships with nature foster contributions to the greater whole by creating relational wealth and conscious well-being based on care, empathy, and respect (Spiller et al., 2011).

#### Multidimensional Regenerative Value

By emphasizing the importance of considering place-based cultural and spiritual dimensions in value creation for the health and well-being of social-ecological systems, this article expands regenerative organizing research beyond the ecological and organizational performance-based dimensions of regeneration (Rahman et al., 2024; Slawinski et al., 2021). This article also expands the scope of stakeholder value toward regenerative value (Konietzko et al., 2023), creating a new theoretical space within stakeholder research that can explain how nature-inclusive stakeholder relationships can benefit both human and non-human nature. Regenerative organizing scholars call for a better understanding of stakeholder engagement for regeneration (Das & Bocken, 2024; Rahman et al., 2024) and an “alternative theoretical lens, capable of capturing and examining the [. . .] interactions through which a regenerative enterprise engages with natural ecosystems” (Muñoz & Hernandez, 2024, p. 579). The stakeholder engagement construct proposed next may represent such a theoretical lens that explains how multidimensional human-nature stakeholder relationships can regenerate social-ecological systems.

#### Redefining Nature-Inclusive Stakeholder Engagement

The empirical insights from the Indigenous eye of the Sierra Nevada in dialogue with the Western eye of nature-inclusive stakeholder engagement enable envisioning what I propose as multidimensional nature-inclusive stakeholder engagement. *Multidimensional nature-inclusive stakeholder engagement embraces a nature-sensitive mindset and relational stakeholder approach to consider the roles, needs, and preferences of nature (beings) that act(s) as (a) stakeholder(s) and guide(s) in organizational activity. Profound physical, emotional, mental, and spiritual nature relationships of and interactions by organization-internal, nature-connected human proxies enable the creation of regenerative value.*

The proposed construct highlights four conceptual advances. First, “multidimensional” refers to nature stakeholder relationships that extend physical interactions, including emotional, mental, and spiritual engagement. This complex set of relationships suggests a more comprehensive and inclusive approach to stakeholder engagement that acknowledges a pluriverse of overlapping ontologies and place-based worlds of human-nature relations (Banerjee, 2022; Ehrnström-Fuentes & Böhm, 2023; Gulari et al., 2025). Second, multidimensional nature-inclusive stakeholder engagement includes and perceives nature beings with their non-human characteristics, omitting the anthropomorphizing of nature stakeholders (i.e., not treating non-human beings like other humans; Muñoz & Hernandez, 2024). Third, the proposed construct emphasizes the relational stakeholder view (Post et al., 2002), recognizing the interconnectedness and interdependence of heterogeneous stakeholder relationships, as well as the normative stakeholder view, which considers the intrinsic value of stakeholders (Donaldson & Preston, 1995). These views align with regenerative organizing as a relational process, dependent on including a wide range of co-dependent stakeholders (Robinson & Cole, 2015). Finally, the construct has implications for stakeholder research that deals with nature, calling for a profound organizational understanding of the complex interrelation between nature as a unified stakeholder and particularized nature stakeholders, including humans and their related stakeholder relationships, thereby opening up theoretical space for systems thinking (Bansal et al., 2018).

### Managerial Implications of Multidimensional Nature-Inclusive Stakeholder Engagement

Despite the findings’ limited generalizability to other distinct (Indigenous) business contexts (Alcadipani et al., 2012; Weston & Imas, 2018), engaging in co-learning guided by Two-Eyed Seeing (Bartlett et al., 2012) suggests managerial implications beyond the studied Indigenous businesses, as introduced next through reflexive questions for organizational representatives.

#### How can Considering Nature Stakeholder Relationships Beyond Physical Interactions Shape the Organization-Nature Connection for Regenerative Value Creation?

Embracing multidimensional relationships with nature stakeholders, encompassing physical, emotional, mental, and spiritual interactions, enables organizational representatives to engage with nature stakeholders with greater sensitivity through a strengthened self-reflective connection to nature (Connolly & Cullen, 2018; Driscoll & Starik, 2004; Sama et al., 2004). This sensitivity, obtained from acquiring and combining knowledge gained through these multidimensional relationships and dedicating time and space to exploring them, can yield nature-based innovations and solutions (Hart & Sharma, 2004; Jacobs, 1997). In addition, the enhanced understanding of nature stakeholder relationships allows managers to untangle the complexity of how an organization affects nature stakeholders and how the latter affect the organization and its stakeholders (Heikkinen et al., 2019; Kujala et al., 2019). However, managers should be aware of the limited manageability of value creation contexts involving nature stakeholders. The notion of nature (stakeholders) as (a) guide(s) highlights that nature can never be fully controlled. Gulari et al. (2025) argue that nature relationships that go beyond extractivism are complex, asking that managers deal with rather than seek to reduce complexity. Hence, multidimensional nature-inclusive stakeholder engagement remains a dynamic managerial approach that may broaden organizational ethical considerations of stakeholders to encompass not only those in traditional positions of power (Tallberg et al., 2024).

#### Which Representation of Nature (Beings) as (a) Stakeholder(s) Allows for an Adequate Consideration of its (their) Roles, Needs, and Preferences in Value-Creation Activities?

The proposed multidimensional nature-inclusive stakeholder engagement construct illustrates how a more direct representation of nature in business activity can unfold, in contrast to its representation through third-party human proxies. Organization-external intermediaries are often incapable of or face conflicts of interest in adequately representing nature stakeholders’ “stakes,” instead drawing attention to themselves and creating unfavorable organizational dependencies (Driscoll & Starik, 2004; Gulari et al., 2025; Kortetmäki et al., 2023). Relying on such proxies for nature (stakeholders) becomes especially problematic when their worldviews conflict with or do not fully embrace the focal organization’s understanding of what constitutes nature and how to interact culturally adequately, such as in Indigenous business settings (Love, 2020). In contrast, nature-connected organization-internal human proxies for nature (stakeholders) can more adequately and comprehensively reflect on the organizational coexistence and interrelatedness with nature (stakeholders) by relying on internal organizational information and place-based knowledge, and nature interactions (Hart & Sharma, 2004; Rahman et al., 2024; Tryggestad et al., 2013).

#### How can the Needs and Preferences of Nature Stakeholders be Adequately Voiced?

Multidimensional nature-inclusive stakeholder engagement visibilizes nature stakeholders within organizations by systematically incorporating them into managerial thinking and decision-making (Connolly & Cullen, 2018; Laine, 2010; Starik, 1995). However, the representation of nature stakeholders by organization-internal, nature-connected human proxies does not address scholars’ concerns about how to correctly voice nature stakeholders’ needs (i.e., sustaining climate-specific conditions) and preferences (i.e., being surrounded by particular species) (Blount & Conklin, 2023; Tallberg et al., 2024). Linked to this concern, managers must also be conscious of nature’s wildness and, at times, cruelty, when relying on the proposed stakeholder engagement construct. This consciousness may increase the complexity of decision-making processes, but it is needed to prevent the romanticization and idealization of nature (stakeholders) (Muñoz & Hernandez, 2024). For instance, to create regenerative value, purposeful detachment from or non-interference with nature (stakeholders) must become part of an organization’s attentive care for the latter (Ehrnström-Fuentes et al., 2025; Muñoz & Hernandez, 2024; Sage, 2025). Adhering to business and ecological ethics combined with scientific approaches that involve assessing the biological balance (e.g., the planetary boundaries framework; Lahneman et al., 2025) may provide managerial guidance for countering the uncertainty surrounding “humans’ subjective evaluation of what is best for these animal or environmental stakeholders” (Blount & Conklin, 2023, p. 262). Furthermore, this article encourages organizational representatives to rely on their human ability “to listen to, or to know about, the needs, interests, and stakes of nonhumans” (Gulari et al., 2025, p. 232), which can be strengthened by creating organization-specific knowledge through multidimensional nature (stakeholder) relationships.

### Limitations and Areas for Future Research

This article is not without its limitations, which may orient future research endeavors. First, I am aware that critics will argue that my proposed nature-inclusive stakeholder engagement construct is the result of Western reasoning and informs Western capitalist theory. I agree, while emphasizing the adoption of the analytical lens of Two-Eyed Seeing for the conceptualization of the proposed construct, which helps respectfully engage with Indigenous knowledge systems (Bartlett et al., 2012). In addition, I acknowledge that, to address systemic challenges, related established theories and assumptions need to be re-evaluated by becoming spaces for dialogue between non-Western and Western perspectives that generate new imaginaries. Hence, I encourage organizational and management scholars to engage with Two-Eyed Seeing (Bartlett et al., 2012) and to conduct decolonizing research to re-evaluate the predominantly extractive and transactional Western organization-nature relationship.

Second, I conducted the interviews with the Indigenous research participants in Spanish. As Indigenous languages cannot be easily translated into other languages (Schneider & Kayseas, 2018), my approach limited the understanding of the studied Indigenous cosmovision, ontologies, and culture to a certain degree. I tried to avoid “erasing the holistic foundation of Indigenous knowledges” (Banerjee, 2022, p. 1081) as far as possible through my decolonizing research approach. However, further research on Indigenous organizing in the Sierra Nevada may rely on Indigenous language assistants to ensure the greatest accuracy of collected data. Moreover, when Indigenous researchers can be identified in the studied Indigenous setting, research collaboration should be targeted.

Third, my article provides limited insights into how the community-based structures of the studied businesses impact regenerative value creation, in addition to the identified mindset and interactions. While this article adopts a firm-centric lens to analyze organization-nature relationships, adopting a community-oriented lens (Arjaliès & Banerjee, 2024; Fan, 2024) can shed further light on how businesses address collective interests (Peredo, 2019, 2023; Peredo & Chrisman, 2006). For example, studying community-based businesses through a stakeholder network lens (Schneider & Sachs, 2017) may help explain how regenerative value unfolds across organizations, communities, and natural ecosystems.

Finally, the proposed organization-internal human proxy approach of representing nature suggests an exciting and promising avenue for organizational and management research into self-representational issues concerning nature stakeholders. This article creates the ground for studying *who* should have the right to represent nature and the limitations thereof, advancing research streams such as marginalized stakeholder theory (Chowdhury, 2021). Moreover, studying *when* and *how* nature beings become salient stakeholders for organization-internal human proxies may further strengthen the managerial applicability of multidimensional nature-inclusive stakeholder engagement.

## Conclusion

The multidimensional nature-inclusive stakeholder engagement advanced in this article contributes to scholarly efforts to realign organization-nature relationships to address nature’s increasing degradation (Haigh & Griffiths, 2009; Kortetmäki et al., 2023). The identified mindset, related interactions, and regenerative value types based on the empirical insights delivered by the Indigenous eye of the Sierra Nevada shift the rather theoretical-philosophical debate on nature as a stakeholder toward a more refined managerial approach. Adopting a decolonizing research approach, including Two-Eyed Seeing, the resulting nature-inclusive stakeholder engagement construct emphasizes a “co-creative partnership with nature” (du Plessis, 2012, p. 19). A nature-sensitive organizational mindset that embraces nature (beings) as (a) guide(s) and stakeholder(s) characterizes this partnership. Organization-internal, nature-connected human proxies who follow a relational stakeholder approach and embrace physical, emotional, mental, and spiritual relationships with nature stakeholders put this partnership into practice for multidimensional regenerative value creation. Hence, this article illustrates how respectfully engaging with the millennia-old cosmovisions, knowledges, and experiences of Indigenous peoples can contribute to further challenging anthropocentric and Western biases in management and organizational research and practice that impede theorization and action associated with a more harmonious relationship between humans and other nature beings (Ehrnström-Fuentes et al., 2025; Ehrnström-Fuentes & Böhm, 2023; Love, 2020; Tallberg et al., 2024). As is evident from this article, such a relationship can yield a net-positive impact on society and nature instead of degrading, depleting, and destroying the foundation of all life and that of most businesses’ value creation: (Mother) Nature.

## Appendix 1

**Table A1.** Leading Semi-Structured Interview Questions (Translated to English).

**First round of interviews**
What is your relationship with nature in your day-to-day business?
Which nature beings are relevant to your business activities and why?
How would you describe your relationships with these nature beings in a typical workday?
How do you interact with these nature beings in your business activities?
How do you include nature beings relevant to your business in planning and other processes in your day-to-day business?
**Second round of interviews**
In your work, why is it essential to include the interests and needs of different nature beings?
What factors influence your attitude toward different nature beings in your work, and how?
How would you describe this attitude toward nature beings in your work?
What interactions with different nature beings influence your work, and how?
What are the costs/benefits of poor/adequate interactions with nature beings in your business context?

## Notes

1. All interview quotations are translations by the author.
